# Supplementary material for: qPCR multiplex detection of microRNA and messenger RNA in a single reaction
Source: PeerJ. 2020 Jun 25;8:e9004. doi: 10.7717/peerj.9004 (PMC7321665; doi:10.7717/peerj.9004)
Supplement: Table S1 [file peerj-08-9004-s001.doc]

| **Sample I.D** | **Cell Line HEK 293** | | | | | | | |
| --- | --- | --- | --- | --- | --- | --- | --- | --- |
| *Replicate 1* | Concentration (ng/µl) | A260 | | A280 | | | 260/280 | 260/230 |
|  | 9403.583 | 235.09 | | 113.229 | | | 2.076 | 1.722 |
| *Replicate 2* | 8713.994 | 217.85 | | 104.831 | | | 2.078 | 1.692 |
| *Replicate 3* | 8699.546 | 217.489 | | 104.801 | | | 2.075 | 1.683 |
|  | **Transfection HeLa Cell Line** | | | | | | | |
| *Sample 1 Replicate 1* | Concentration (ng/µl) | | A260 | A280 | | 260/280 | | 260/230 |
|  | 110.13 | | 2.753 | 1.342 | | 2.05 | | 2.12 |
| *Sample 1 Replicate 2* | 104.41 | | 2.61 | 1.272 | | 2.05 | | 2.09 |
| *Sample 1 Replicate 3* | 104.51 | | 2.613 | 1.247 | | 2.09 | | 2.1 |
| *Sample 2 Replicate 1* | 107.85 | | 2.696 | 1.317 | | 2.05 | | 2.11 |
| *Sample 2 Replicate 2* | 108.19 | | 2.705 | 1.317 | | 2.05 | | 2.07 |
| *Sample 2 Replicate 3* | 108.8 | | 2.72 | 1.345 | | 2.02 | | 2.05 |
| *Sample 3 Replicate 1* | 278.12 | | 6.953 | 3.393 | | 2.05 | | 2.18 |
| *Sample 3 Replicate 2* | 278.47 | | 6.962 | 3.407 | | 2.04 | | 2.15 |
| *Sample 3 Replicate 3* | 281.16 | | 7.029 | 3.426 | | 2.05 | | 2.18 |
| *Sample 4 Replicate 1* | 204.42 | | 5.11 | 2.495 | | 2.05 | | 2.13 |
| *Sample 4 Replicate 2* | 205.04 | | 5.126 | 2.509 | | 2.04 | | 2.13 |
| *Sample 4 Replicate 3* | 205.87 | | 5.147 | 2.492 | | 2.07 | | 2.15 |
|  | **Human Serum** | | | | | | | |
| *Sample 1 Replicate 1* | Concentration (ng/µl) | A260 | | | A280 | 260/280 | | 260/230 |
|  | 10.154 | | 0.254 | | 0.183 | 1.386 | | 0.256 |
| *Sample 1 Replicate 2* | 10.246 | | 0.256 | | 0.192 | 1.335 | | 0.258 |
| *Sample 1 Replicate 3* | 9.276 | | 0.232 | | 0.169 | 1.375 | | 0.242 |
| *Sample 2 Replicate 1* | 19.061 | | 0.452 | | 0.337 | 1.341 | | 0.42 |
| *Sample 2 Replicate 2* | 17.176 | | 0.429 | | 0.325 | 1.322 | | 0.411 |
| *Sample 2 Replicate 3* | 17.385 | | 0.435 | | 0.318 | 1.367 | | 0.406 |
| *Sample 3 Replicate 1* | 23.327 | | 0.583 | | 0.437 | 1.335 | | 0.322 |
| *Sample 3 Replicate 2* | 21.495 | | 0.537 | | 0.407 | 1.321 | | 0.319 |
| *Sample 3 Replicate 3* | 19.403 | | 0.485 | | 0.359 | 1.35 | | 0.31 |
| *Sample 4 Replicate 1* | 17.422 | | 0.436 | | 0.3 | 1.451 | | 0.45 |
| *Sample 4 Replicate 2* | 17.626 | | 0.441 | | 0.301 | 1.465 | | 0.446 |
| *Sample 4 Replicate 3* | 17.201 | | 0.43 | | 0.288 | 1.495 | | 0.443 |

**Supplemental Table 1: RNA Concentration and Quality**
