# Supplementary material for: qPCR multiplex detection of microRNA and messenger RNA in a single reaction
Source: PeerJ. 2020 Jun 25;8:e9004. doi: 10.7717/peerj.9004 (PMC7321665; doi:10.7717/peerj.9004)
Supplement: Table S2 [file peerj-08-9004-s002.doc]

**Supplemental Table 2: First Strand cDNA Synthesis Cycle Conditions**

|  | **Program the thermal cycler using any of the three conditions below.** | | | |
| --- | --- | --- | --- | --- |
|  | **Step 1** | **Step 2** | **Step 3** | **Step 4** |
| 1. **MicroRNA synthesis** | | | | |
| Temperature | 16 ºC | 42 ºC | 85 ºC | 4 ºC |
| Time | 30 min | 30 min | 5 min | ∞ |
| 1. **Coding Gene synthesis** | | | | |
| Temperature | 25 ºC | 37 ºC | 85 ºC | 4 ºC |
| Time | 10 min | 120 min | 5 min | ∞ |
| 1. **RNAmp synthesis** | | | | |
| Temperature | 25 ºC | 37 ºC | 85 ºC | 4 ºC |
| Time | 10 min | 120 min | 5 min | ∞ |
