# Supplementary material for: qPCR multiplex detection of microRNA and messenger RNA in a single reaction
Source: PeerJ. 2020 Jun 25;8:e9004. doi: 10.7717/peerj.9004 (PMC7321665; doi:10.7717/peerj.9004)
Supplement: Table S3 [file peerj-08-9004-s003.doc]

Supplemental Table 3: Reaction mix composition of three RT methods

|  | **Method** | | | | | |
| --- | --- | --- | --- | --- | --- | --- |
|  | MicroRNA synthesis | | Coding Gene synthesis | | RNAmp synthesis | |
| **Components** | Volume (µl) | Final Concentration | Volume (µl) | Final Concentration | Volume (µl) | Final Concentration |
| 10× RT Buffer, 1.0ml | 1.5 | 1× | 2.0 | 1× | 1.5 | 1× |
| MgCl2 25mM, 1.5ml | - | - | - | - | 0.5 | 0.83mM |
| 25× dNTP Mix 100mM, 200 μL | 0.15 | 1mM | 0.8 | 4mM | 0.25 | 1.6mM |
| RNase Inhibitor 100μL, 20Units/μL) | 0.2 | 4 U | 1.0 | 20 U | 0.2 | 4 U |
| RNA Input (100ng/μL) | 1 | - | 1 | - | 1 | - |
| 5× miRNA RT Primer | 6μL total | - | - | - | 6μL total | - |
| 10× RT Random Primer, 1.0ml | - | - | 2.0 | 1× | 1.5 | 1× |
| MultiScribe™ Reverse Transcriptase 100 μL, 50Units/μL | 1.0 | 50 U | 1.0 | 50 U | 1.0 | 50 U |
| Water | 5.15 | - | 12.2 | - | 3.05 | - |
|  | **15.0 μL Final Volume** | | **20.0 μL Final Volume** | | **15.0 μL Final Volume** | |
