# Supplementary material for: qPCR multiplex detection of microRNA and messenger RNA in a single reaction
Source: PeerJ. 2020 Jun 25;8:e9004. doi: 10.7717/peerj.9004 (PMC7321665; doi:10.7717/peerj.9004)
Supplement: Table S4 [file peerj-08-9004-s004.doc]

**Supplemental Table 4: Reaction mix composition of singleplex qPCR reaction**

|  | **Reaction Component** | | | | | | | |
| --- | --- | --- | --- | --- | --- | --- | --- | --- |
| Singleplex | Volume (µl) | Final Concentration | Volume (µl) | Final Concentration | Volume (µl) | Final Concentration | Volume (µl) | Final Concentration |
| Taqman Assay (20×) | 0.5 | 0.5× | 0.5 | 1× | 0.5 | 2× | 0.5 | 4× |
| Taqman Universal PCR Master Mix (2×) | 10.0 | 1× | 5.0 | 1× | 2.5 | 1× | 1.0 | 0.8× |
| cDNA input | 1.0 | - | 1.0 | - | 1.0 | - | 1.0 | - |
| Water | 8.5 | - | 3.5 | - | 1.0 | - | - | - |
|  | **20.0 μL Final Volume** | | **10.0 μL Final Volume** | | **5.0 μL Final Volume** | | **2.5 μL Final Volume** | |
