# Supplementary material for: qPCR multiplex detection of microRNA and messenger RNA in a single reaction
Source: PeerJ. 2020 Jun 25;8:e9004. doi: 10.7717/peerj.9004 (PMC7321665; doi:10.7717/peerj.9004)
Supplement: Table S5 [file peerj-08-9004-s005.doc]

**Supplemental Table 5. Reaction Mix composition of duplex qPCR reactions**

|  | **Reaction Component** | | | | | | | |
| --- | --- | --- | --- | --- | --- | --- | --- | --- |
| Duplex (2 multi-labelled Targets) | Volume (µl) | Final Concentration | Volume (µl) | Final Concentration | Volume (µl) | Final Concentration | Volume (µl) | Final Concentration |
| TaqMan Assay (20×) | 0.5 each | 0.5× each | 0.5 each | 1× each | 0.5 each | 2× each | 0.5 each | 4× each |
| TaqMan Universal PCR Master Mix (2×) | 10.0 | 1× | 5.0 | 1× | 2.5 | 1× | 1.0 | 0.8× |
| cDNA input | 1.0 | - | 1.0 | - | 1.0 | - | 1.0 | - |
| Water | 8.0 | - | 3.0 | - | 0.5 | - | - | - |
|  | **20.0 μL Final Volume** | | **10.0 μL Final Volume** | | **5.0 μL Final Volume** | | **2.5 μL Final Volume** | |

**Supplemental Table 5b:**

**Probes and dye layers for Figures 4.1 to 4.4**

| **Figure 4.1** | | **Figure 4.2** | | **Figure 4.3** | | **Figure 4.4** | |
| --- | --- | --- | --- | --- | --- | --- | --- |
| Probe | Dye | Probe | Dye | Probe | Dye | Probe | Dye |
| ACTB | VIC | 18S | VIC | 18S | VIC | JAG1 (Duplex) | FAM |
| B2M | VIC | GAPDH | VIC | ACTB | VIC | hsa-miR-miR21 (Duplex) | VIC |
| hsa-miR-21 | VIC | ACTB | VIC | GAPDH | VIC | hsa-miR-486-5p | FAM |
| hsa-miR-99b | FAM | P53 | FAM | B2M | VIC | hsa-miR-451 | FAM |
| hsa-miR-16 | FAM | U75 | FAM | CDKN1 | FAM | B2M | VIC |
| U75 | FAM | Let-7c | FAM | P53 | FAM | - | - |
| - | - | hsa-miR-99b | FAM | P16 | FAM | - | - |
| - | - | hsa-miR-21 | VIC | Dicer | FAM | - | - |
| - | - | - | - | Ago2 | FAM | - | - |
