# Supplementary material for: qPCR multiplex detection of microRNA and messenger RNA in a single reaction
Source: PeerJ. 2020 Jun 25;8:e9004. doi: 10.7717/peerj.9004 (PMC7321665; doi:10.7717/peerj.9004)
Supplement: Table S6 [file peerj-08-9004-s006.doc]

**Supplemental Table 6: The change in quantification cycle (Cq) values for selected mRNA, miRNAs in smaller fluorescent qPCR reaction volumes.** There was an overall decrease in Cq mean values for all amplicons suggesting an increase in detection sensitivity at lower reaction volumes.

|  | **Actin** | | **B2M** | |
| --- | --- | --- | --- | --- |
| Volume μl | *Mean* | *SEM* | *Mean* | *SEM* |
| 20.0 | 27.02 | 0.420 | 27.08 | 0.816 |
| 10.0 | 22.87 | 0.251 | 23.86 | 0.600 |
| 5.0 | 20.33 | 0.248 | 22.36 | 0.340 |
| 2.5 | 17.77 | 0.138 | 20.66 | 0.766 |
|  |  |  |  |  |
|  | **Hsa-miR-21** | | **Hsa-miR-99b** | |
| Volume μl | *Mean* | *SEM* | *Mean* | *SEM* |
| 20.0 | 25.48 | 0.189 | 26.13 | 0.196 |
| 10.0 | 23.15 | 0.067 | 24.61 | 0.260 |
| 5.0 | 21.74 | 0.155 | 23.00 | 0.161 |
| 2.5 | 18.93 | 0.089 | 21.67 | 0.349 |
|  |  |  |  |  |
|  | **Serum hsa-miR-16** | |  |  |
| Volume μl | *Mean* | *SEM* |  |  |
| 20.0 | 22.00 | 0.496 |  |  |
| 10.0 | 20.07 | 0.200 |  |  |
| 5.0 | 19.24 | 0.167 |  |  |
| 2.5 | 16.98 | 0.259 |  |  |
