# Supplementary material for: qPCR multiplex detection of microRNA and messenger RNA in a single reaction
Source: PeerJ. 2020 Jun 25;8:e9004. doi: 10.7717/peerj.9004 (PMC7321665; doi:10.7717/peerj.9004)
Supplement: Table S7 — Fluorophores with narrow, well-resolved bandwidths are useful for duplex qRT-PCR applications avoiding cross talk. ROX™ is the passive reference utilised in a Step One Plus. [file peerj-08-9004-s007.docx]

| **Dye** | **Ab_max_ (nm)** | **Extinction Coefficient (l mole–1 cm–1)** | **Em_max_ (nm)** |
| --- | --- | --- | --- |
| ROX™ | 585 | 82,000 | 605 |
| VIC® | 538 | NA | 554 |
| 6-FAM™ | 494 | 83,000 | 518 |

**Supplemental Table 7: Common Fluorophore bandwidths.** Fluorophores with narrow, well-resolved bandwidths are useful for duplex qRT-PCR applications avoiding cross talk. ROX™ is the passive reference utilised in a Step One Plus.
